# Treatise on Spermatorrhœa, Impotence, and Sterility

**Published:** 1897-09

**Authors:** 


Treatise on Spermatorrhœa, Impotence, and Sterility. By William Harvey King, M. D., Professor of Electro-Therapeutics in the New York Homœopathic College, Metropolitan Post-Graduate School of Medicine, and the New York College and Hospital for Women. 178 pages. New York: A. C. Chatterton & Co., 133 William St. 1897. Price, bound in cloth, post paid, $1.50.

This important book treats of the sexual diseases of men of a functional character from the standpoint of the physician and not that of the surgeon.

The author is a specialist in electricity, and his principle of treatment is, therefore, electrical. Nevertheless, he gives a few indications for homœopathic remedies. He has evidently had much opportunity to observe this kind of cases. His hygienic management of them is admirable, and will lead to frequent consultations of the book in treating this class of cases. As the author well says in his preface, the hygienic management is "the keynote of the successful treatment of a large proportion of such cases."

The authorities quoted by the author are Gross, Ultzman, and Milton.

At best these conditions are hard to treat, and it is desirable to have concise works of reference where available hints may be obtained for treatment. These hints, where they embrace hygiene, are good, those of a medicinal character are not as good as they might be, while those of an electrical character are still less valuable. However, it is not to the failings of the book it is our object to draw attention, but to its excellencies.

